# Shotgun proteomics for biomarker discovery in microbial pathogens and allergens in food: a review

**DOI:** 10.3389/fbioe.2026.1675503

**Published:** 2026-04-08

**Authors:** Manuel G. Amado, Paula Borrajo, Manuel Pazos, Jorge Barros-Velázquez, Pilar Calo-Mata, Ana G. Abril, Mónica Carrera

**Affiliations:** 1 Department of Food Technology, Institute of Marine Research (IIM-CSIC), Spanish National Research Council (CSIC), Vigo, Spain; 2 Advanced Omics Service, Institute of Marine Research (IIM-CSIC), Spanish National Research Council (CSIC), Vigo, Spain; 3 Department of Analytical Chemistry, Nutrition and Food Science, School of Veterinary Sciences, University of Santiago de Compostela (USC), Lugo, Spain; 4 Department of Functional Biology and Health Science, Faculty of Biology, University of Vigo (UVIGO), Vigo, Spain

**Keywords:** biomarker, food allergen, foodborne pathogenic bacteria, LC-MS/MS, protein identification, protein quantification, shotgun proteomics, virulence factors

## Abstract

Shotgun proteomics has revolutionized protein research by combining liquid chromatography with tandem mass spectrometry (LC-MS/MS), enabling the identification and quantification of proteins in complex mixtures. This technique has significantly advanced the study of various foodborne pathogenic bacteria and food allergens rapidly and robustly. This review article compiles the use of shotgun proteomics for characterizing key biomarkers in foodborne pathogenic bacteria, facilitating their specific identification and the characterization of virulence factors, antibiotic resistance, and other biomarkers. These biomarkers are crucial for investigating the most relevant foodborne pathogens that contaminate food intended for human consumption. Additionally, the review will cover current applications of proteomics in identifying food allergens, aiming to prevent such hazards and protect consumers. Furthermore, new trends in shotgun proteomics will be discussed to highlight the most recent advances in the field.

## 1 Introduction

Food safety is a major concern worldwide due to the health risks associated with the presence of microbial pathogens and allergens in food products. The global increase in foodborne illnesses and allergic reactions highlights the urgent need for accurate, rapid, and sensitive detection methods to ensure food quality and protect public health (Devleesschauwer et al., 2015). Traditional microbiological and immunological techniques, although widely used, often require long processing times and may lack the sensitivity or specificity needed for the identification of emerging pathogens and allergens (Law et al., 2015).

In this context, proteomics has emerged as a powerful tool for food safety applications, offering detailed insights into the protein composition of complex biological samples (Aebersold and Mann, 2016). Among the different proteomics strategies, shotgun proteomics—based on the combination of liquid chromatography with tandem MS (LC-MS/MS)—has gained significant attention for its capacity to simultaneously identify and quantify thousands of proteins in a given biological system (Zhang et al., 2013).

In a typical shotgun proteomics workflow, a complex mixture of proteins is enzymatically digested, usually with a protease such as trypsin, generating a heterogeneous mixture of peptides. These peptides are then separated by single or multiple rounds of LC and subsequently analyzed by MS using different fragmentation techniques (Domon and Aebersold, 2010; Carrera and Mateos, 2021). Bioinformatics tools and database search algorithms are employed to validate the peptide sequences and assign them to their corresponding proteins (Nesvizhskii, 2010). This strategy has revolutionized the field of proteomics, as it allows the high-throughput identification and quantification of thousands of proteins in a single analysis. This high-throughput approach enables a comprehensive analysis of food matrices, microbial contaminants, and allergenic components with unprecedented depth and precision, offering new opportunities for improving food safety and quality monitoring (Carrera and Mateos, 2021).

Shotgun proteomics has proven particularly valuable for the characterization of biomarkers associated with foodborne pathogenic bacteria, including virulence factors, antibiotic resistance proteins, and species-specific markers (Abril et al., 2024a; Abril et al., 2025). These biomarkers not only facilitate pathogen detection but also contribute to a better understanding of microbial behavior and pathogenicity mechanisms. Furthermore, shotgun proteomics has advanced the identification of food allergens by allowing the direct detection of allergenic proteins in complex food matrices, overcoming some of the limitations associated with conventional immunoassays (Carrera and Mateos, 2021).

This review aims to provide an overview of the current applications of shotgun proteomics for the identification and characterization of biomarkers relevant to the detection of microbial pathogens and allergens in food. By compiling recent advances and highlighting future perspectives, we aim to underline the importance of integrating shotgun proteomics into food safety monitoring strategies and allergen risk management.

## 2 Shotgun proteomics for identification and quantification of proteins

Shotgun proteomics is one of the most widely used strategies for the comprehensive identification and quantification of proteins in complex biological systems. In this bottom-up proteomics approach, proteins are first enzymatically digested into peptides, typically using trypsin, which cleaves proteins at the carboxyl side of lysine and arginine residues. The resulting peptide mixture is then subjected to one-dimensional (1D) or two-dimensional (2D) LC separation to reduce sample complexity before being analyzed by MS/MS (Aebersold and Mann, 2016).

MS plays a central role in shotgun proteomics, offering high sensitivity, accuracy, and resolution for peptide detection and sequencing (Shuken, 2023). Various fragmentation techniques, such as collision-induced dissociation (CID), higher-energy collisional dissociation (HCD), or electron transfer dissociation (ETD), are traditionally employed. More recently, ultraviolet photodissociation (UVPD) has emerged as a highly efficient fragmentation method that generates extensive peptide fragmentation, improving sequence coverage and enhancing the identification of post-translational modifications (PTMs) (Brodbelt, 2014).

Data acquisition strategies in shotgun proteomics have also evolved significantly. Data-dependent acquisition (DDA) was the conventional method, where the most intense precursor ions are sequentially selected for fragmentation. However, DDA is inherently biased towards abundant peptides. To overcome this, data-independent acquisition (DIA) strategies, such as SWATH-MS, have been developed, where all ions within a selected *m/z* window are fragmented in parallel, enabling comprehensive and reproducible peptide identification and quantification across multiple samples (Gillet et al., 2012).

Quantification in shotgun proteomics can be achieved through label-free methods or labeling strategies. Label-free quantification relies on comparing peptide ion intensities or spectral counting, offering a straightforward and cost-effective approach (Guzman et al., 2024). Alternatively, labeling strategies such as tandem mass tags (TMT) (Stryiński et al., 2019), isobaric tags for relative and absolute quantification (iTRAQ), or stable isotope labeling by amino acids in cell culture (SILAC) enable multiplexed and highly accurate quantitative analysis (Ong et al., 2002).

Recent advances in MS instrumentation, including improvements in mass accuracy, resolution, sensitivity, and scanning speed, have significantly enhanced the performance of shotgun proteomics workflows (Shuken, 2023). Instruments such as quadrupole-Orbitraps, time-of-flight (TOF) and ion mobility analyzers now allow deep proteome profiling, facilitating the detection of low-abundance proteins and providing comprehensive insights into complex biological systems.

In parallel, the integration of multiomics approaches—combining proteomics with genomics, transcriptomics, metabolomics, and lipidomics—has expanded the systems biology perspective, enabling a more holistic understanding of biological functions and disease mechanisms (Babu and Snyder, 2023). In food science, this integration helps characterize microbial contamination, allergen presence, and food matrix changes more thoroughly.

Moreover, imaging MS techniques, such as matrix-assisted laser desorption/ionization (MALDI) imaging, have been adapted to shotgun proteomics workflows, allowing the spatial distribution of proteins and peptides to be visualized directly in tissues or food samples without the need for labeling (Moore and Charkoftaki, 2023). This approach opens new possibilities for localizing pathogenic microorganisms or allergenic proteins in complex food matrices (Feucherolles et al., 2022).

The application of machine learning (ML) and artificial intelligence (AI) is also transforming shotgun proteomics. These technologies are increasingly used to optimize data acquisition, improve peptide and protein identification rates, predict fragmentation patterns, and enhance quantitative accuracy (Mann et al., 2021). Deep learning algorithms, such as those implemented in tools like Prosit and DeepMass, have revolutionized spectrum prediction, leading to more accurate database searches and increasing proteome coverage (Gessulat et al., 2019). In particular, the combination of ML and DIA data analysis has proven highly powerful for extracting meaningful biological information from complex datasets. ML models can assist in biomarker discovery, quality control, and the prediction of protein–protein interactions, facilitating the translation of proteomics findings into actionable knowledge for food safety applications (Tran et al., 2017).

Despite these advancements, shotgun proteomics faces ongoing challenges, including dynamic range limitations, missing data, and the complexity of interpreting large datasets. Continuous improvements in sample preparation, instrumentation, data analysis algorithms, and integrative omics approaches are critical to overcoming these barriers and advancing the field toward achieving comprehensive proteome characterization at both qualitative and quantitative levels.

In conclusion, shotgun proteomics stands at the forefront of protein science, driven by rapid technological innovations and the integration of artificial intelligence, machine learning, and multiomics strategies. These developments are pushing the boundaries of deep proteome analysis and opening new horizons for applications in food safety, pathogen detection, and allergen monitoring.

## 3 Shotgun proteomics for foodborne pathogenic bacteria characterization

Given the paramount importance of foodborne pathogenic bacteria to human health and wellness, and the importance to ensure high-quality safe foodstuff, the characterization of food spoiling microorganism with the aim of avoiding several illnesses is crucial. Over the past decades, outbreaks of foodborne illnesses have underscored the need to develop and implement preventive measures to ensure food safety (Riwu et al., 2022). Hence, the detection and characterization of foodborne microorganisms has become a research hotspot. The proteomics approach is an emerging technology that facilitates the characterization of microorganisms attending to their proteome variations, thereby aiding the detection of different foodborne pathogens and spoilage microorganisms (Afzaal et al., 2022).

Virulence factors are specific molecules, mainly proteins (enzymes, glycoproteins and lipoproteins) secreted by different pathogenic microorganisms, including bacteria, viruses, fungi, and protozoa, to enhance their pathogenicity. These factors are related to disease development and resistance to antibiotics, and they are encoded by specific genes localized within the bacterial chromosome or on mobile genetic elements, such as plasmids and transposons. Besides the aforementioned proteins, other molecules are also produced, such as toxins, exopolysaccharides, and cell surface structures like lipopolysaccharides and capsules. Furthermore, modifications in metabolic regulatory networks, mediated by protein sensors and non-coding regulatory RNAs, have also been recognized as integral factors in the modulation of microbial virulence (Leitão, 2020; Pakbin et al., 2021b).

Bacterial foodborne diseases result from infections caused by various pathogenic bacteria, including *Campylobacter* spp., *Escherichia coli*, *Klebsiella* spp., *Pseudomona*s spp., *Salmonella*, *Shigella* spp., *Vibrio* spp., and *Listeria monocytogenes*, among others. After the ingestion of these pathogens, different symptoms appear, leading to foodborne infections such as salmonellosis and listeriosis, or through microbial toxins produced within the host, resulting in toxic infections like *Clostridium perfringens* food poisoning. Major outbreaks have been frequently associated with dairy products, fruits, ground meat, poultry, seafood and vegetables (Liu et al., 2022). The characterization of pathogens isolated from food that have caused infections in individuals will allow for the rapid identification of the most effective antimicrobial for each pathogen.

**FIGURE 1:**
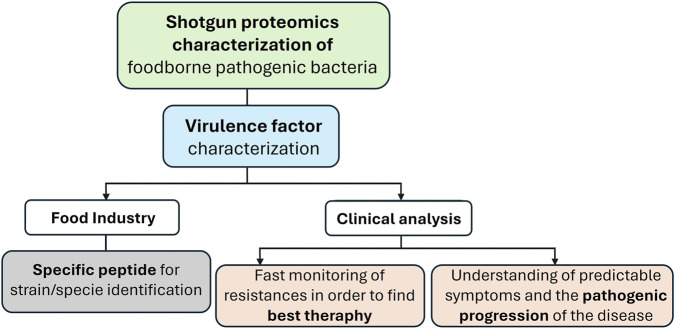
This flowchart illustrates the advancements in shotgun proteomics and the insights derived from its application to the study of foodborne pathogens. This methodology enables precise strain identification, rapid detection of antibiotic resistance, and a deeper comprehension of disease progression, supporting both key application areas: the food industry and clinical diagnostics.

Regarding the methodologies available for pathogen characterization and considering the topic from a critical perspective, shotgun proteomics emerges as a particularly valuable technique in microorganism characterization and identification of virulent factors, due to its ability to identify and quantify thousands of proteins in complex biological samples without prior knowledge of targets, offering broad proteome coverage and high-throughput analysis. This approach provides direct measurement of protein abundance and post-translational modifications, offering functional insights that genomics and transcriptomics cannot capture. In contrast, immunoassays such as ELISA (Enzyme-Linked Immunosorbent Assay) or Western blot provide high sensitivity, specificity, and clinical robustness but with heavily dependence on antibody quality and unsuitability for unbiased discovery because they require predefined targets. Genomic techniques, including next-generation sequencing, offer scalable and cost-effective analysis of DNA and RNA, however, they provide indirect insights into protein abundance without considering complex protein regulation (post-transcriptional modifications, alternative splicing), making them complementary rather than substitutive to proteomic approaches. While nucleic-acid amplification methods can achieve high analytical sensitivity, they are inherently indirect and frequently detect residual DNA from non-viable or inactive microorganisms, which may overestimate microbial presence. In contrast, shotgun proteomics provides direct evidence of protein abundance, enabling the identification of metabolically active populations and delivering functional insights that genomic approaches cannot capture on their own. Moreover, the ability to predict antimicrobial resistance using genomic information has been greatly enhanced through the advancement of bioinformatics tools and the expansion of genetic repositories. Nevertheless, discrepancies among genomic databases such as incomplete annotations, strain-level variability, and differences in curation standards can lead to inaccurate protein predictions and taxonomic assignments (Hadi et al., 2023). This phenomenon has been illustrated for streptomycin resistance in *Salmonella* spp. and *E. coli*, where adjusting phenotypic thresholds improved the alignment between genotypic and phenotypic data (Tyson et al., 2016). However, shotgun proteomics also has significant limitations as it is critically dependent on comprehensive and well-annotated protein sequence databases. Peptide identification requires matching MS/MS spectra to theoretical peptides derived from genome-encoded protein sequences. As emphasized in comparative proteomics studies, selecting between these methods depends on whether the goal is discovery, quantification, or clinical validation. Moreover, reliable microbial identification by shotgun proteomics generally requires moderate biomass levels, often corresponding to approximately 10^4^–10^6^ cells or equivalent protein amounts depending on sample complexity and enrichment strategies. Table 1 summarizes the principal omics approaches such as genomics (Lüth et al., 2018; Anand and Padmanabhan, 2024; Li et al., 2024), transcriptomics (Chen et al., 2022), proteomics (Shevchuk, 2020; Abril et al., 2025), metabolomics (Chen et al., 2022), lipidomics (O’Donnell et al., 2020; Meena et al., 2025), or integrated multiomics (Anand and Padmanabhan, 2024; Li et al., 2024), highlighting their analytical strengths, limitations and areas where further research is needed. Importantly, shotgun proteomics functional resolution represents a major strength of shotgun proteomics in food systems, where understanding microbial activity rather than mere presence is often critical for assessing spoilage, safety, and fermentation processes. These additions emphasize that shotgun proteomics not only complements genomics but, in many contexts, offers a more biologically meaningful assessment of the microbiome in real food matrices. Table 2 displays a comparative overview of omics approaches applied to foodborne bacterial pathogens: strengths, limitations, and knowledge gaps. Figure 1 shows the principal insights derived from shotgun proteomics application in the characterization of foodborne pathogens.

**TABLE 1:** Comparative overview of omics approaches applied to foodborne bacterial pathogens: strengths, limitations, and knowledge gaps.

Omics approach	Most frequently studied pathogens	Type of information provided	Key strengths	Main limitations	Current knowledge gaps	Reference
Genomics	*Salmonella, E. coli, Listeria*	Genetic potential, strain typing	High robustness, standardization, rapid identification	Does not reflect functional activity	Limited insight into condition-specific expression	Lüth et al. (2018), Li et al. (2024), Anand and Padmanabhan, (2024)
Transcriptomics	*Salmonella, Listeria*	Gene expression regulation	Sensitive to environmental changes	Weak correlation with protein abundance	Poor predictive value for phenotype	Chen et al. (2022)
Proteomics (shotgun)	*Salmonella, E. coli*	Global functional protein expression	Direct functional readout	Technical complexity, reproducibility	Underrepresentation of food matrices	Abril et al. (2025)
Proteomics (targeted)	*E. coli, Salmonella*	Precise protein quantification	High sensitivity and specificity	Requires prior knowledge	Lack of standardized targets	Shevchuk, (2020)
Metabolomics	*Listeria, Salmonella*	Metabolic activity and pathways	Closest to phenotype	High variability, annotation limits	Integration with proteomic data	Chen et al. (2022)
Lipidomics	*Vibrio, Listeria*	Membrane composition, stress adaptation	Relevant for environmental resilience	Limited databases	Sparse pathogen coverage	Meena et al. (2025), O’Donnell et al. (2020)
Integrated multi-omics	Mostly *Salmonella*	Systems-level biological insights	Holistic interpretation	Data integration challenges	Very few comparative, standardized studies	Li et al. (2024), Anand and Padmanabhan, (2024)

**TABLE 2:** Main virulence factors of different foodborne pathogenic bacteria identified by MS.

Species	Identification	Virulence factors	References
Gram-negative			
*Campylobacter* spp.			
*C. jenuni*	MALDI-TOF, nLC-MS/MS	OMV-proteins, Tl proteins, T3SS, T6SS, HtrA protease, CadF or FlpA adhesins, CDT, CPS	Tikhomirova et al. (2024); Kreling et al. (2020); Feucherolles et al. (2022); Sałamaszyńska-Guz et al. (2022); Taheri et al. (2019); Tang et al. (2017)
*Escherichia* spp.			
*E. coli*	MALDI-TOF, nLC−MS/MS (TOF)	Colicin, OmpA, RlpA, Sat, Pal, GroEL, HlyA	Fagerquist et al. (2025); Wurpel et al. (2015); Hong et al. (2019)
*Klebsiella* spp.			
*K. pneumoniae*	MALDI-TOF, nLC−MS/MS	Pal, SlyB, OmpA, OmpX, FimaA_2, Stp_3, OsmB, MetQ, OppA_1, YtfQ_1, YtfQ_2, ClpB_2, DnaK_1, GroL_1, HtpG, GroEL, cagA, DnaJ, secA, tufA, tufB, CpxRA (Spy, CpxP, DsbA, DegP, PpiA), PhoPQ (ArnA, ArnB, ArnC, ArnD, ArnT, LpxL, LpxO), HutI, HutU, HutH, HutG, AstB, AstE, A6T8K5	Huang et al. (2015), Illenseher et al. (2025); Magalhães et al. (2017); Fleitas et al. (2024)
*Pseudomonas* spp.			
*P. aeruginosa*	nLC−MS/MS	AmiE, SigX, T2SS, LasA, LasB, piv, rhlA, rhlB, chitinase ChiC, MagB, MagD	Clamens et al. (2017); Couto., et al. (2015), Fléchard et al. (2018); Ahmed et al. (2019)
*Salmonella* spp.			
*S. enterica*	nLC−MS/MS	T3SS (InvJ, PipB2, PrgI, SpiA, SipB, SipC, SipD, SsaD, SsaJ, SsaK, SsaL, SseE, SseL, SspH2), Che-proteins (CheZ, CheY, CheB, Tsr, Trg), flagellar-proteins (FliB, FliL, FliF, FliM, FliC, FlhE, FliT, FlhA, FliK), CpxR, RpoS, RpoE, RpoH, toxins (VapC, HicA, HicB, HigA, HigB, ParD, adhesins (Ag43, BigA, ShdA, FdeC, PilV, SfmA, YqcG, CcdB, ParD)	Ferrer-Navarro et al. (2016); Li et al. (2019b); Saleh et al. (2019); Abril et al. (2025)
*Shigella* spp.			
*S. flexneri*	MALDI-TOF, nLC−MS/MS	TPX, Thioredoxin/glutathione peroxidase, MobC, OsmY, LptA, AepA, Hmp, lysozyme type C, IpaH1.4, IpaH_5, IpaH_7, IpaB, IpaC, OspD1, MxiC, VirA, VirB, VirC	Pettersen et al. (2021); Batalha et al., 2021); Liu et al. (2017)
*Vibrio* spp.			
*V. cholerae*	MS/MS/MS	AnxA1, TcpB, TcpC, TcpD, TcpF, TcpP, TcpQ, CxA, FlgE, Flgk, FlgL, OmpA, OmpU, OmpV, OmpT, OmpW, DnaK, GroEL, HutA, AcfA	Zoued et al. (2021)
*V. parahaemolyticus*	nLC-TOF MS	OmpA, OmpK, OmpU, TolC, FeoB, NrfC, HutA, CorA, ExbB, ScrB, MshA, Tfp, YscC, YscL, ClpV1, VC_A0110, GntR, Hfq, LuxR, rsmA, VpsT)	Yang et al. (2015)
*V. vulnificus*	MALDI-TOF nLC−MS/MS	FlgA, FlgB, FlgE, FlgF, FlgL, FlgK, FlgJ, EF-Tu, MotB, HupA, OmpN, OmpU, Trx, DnaK, DnaJ, ClpB, ProQ	Zhang et al. (2017); Heinz et al. (2022)
Gram-positive			
*Bacillus* spp.			
*B. cereus*	nLC−TOF MS/MS	EntC BC0813, EntFM BC1953, Hbl1 BC3103, HblB BC3102, NheA BC1809, NheB BC1810, BC0576, BC3385, SecY, SecD, SecF, YajC, YidC2, SleB, CwlJ, GerD, GerKA, GerKC, GerSA, GerRA, GerRC, GerIA, GerLC, SpoVAA, HtrC, YaaH, YpeB	Gao et al. (2021)
*B. licheniformis*	nLC−MS/MS	swrB, MotA, MotB, CheA, CheY, CheY-P, FlgE, FlhA, FlhF, FlgL, Hag, FliF, FliG, FliK, FliM, FliY, Spo0A, Spo0F, Spo0B, GerAA, GerAB, GerAC, Spo0F, KapB, SpoVIF, SodF, YqxM	Wang et al. (2020b)
*Listeria* spp.			
*L. monocytogenes*	nLC−MS/MS	MarR, TetR, HiA, LGX, RelE, ParE, PemK, MazF, EssA, EssB, VirB4, Mga, ESAT-6, GtcA, P60, M60, ComGA, ClpX, ClpP, ClpY, InlA, InlB, InlC, InlJ, FlgK, FlgE, FlgL, MotB, Lmo0696, Lmo0706, Lmo072, Lmo1371, Lmo0135, Lmo2196, OpuCA, PbpA, ClpP, LPxTG, Lmo0129, OpuCA, pbpA, ClpP, LLO, LAP, ActA, FruA, PdhB, PrsA	Luciani et al. (2025); Abril et al. (2021); Stincone et al. (2020)
*Staphylococcus* spp.			
*S. aureus*	nLC−MS/MS	ClfA, ClfB, SraP, CodY, SarR, Fur, Rot, Lpl9, AdcA, Nuc, EbpS, Eno, Map_2, PsmB1, PsmB2, Sbi, HlyII, LukF, LukG, LukH, MetQ, PhnD, PstS, OppA, OpuCC, PrsA, Lpl14, YidC, BdbD, PsmA1-PsmA4, Hld, HlyII, SigB, SpoVG, CcpA, CtsR, FmtA, AgrA, Rot, SbnA, SbnC, SbnE, SbnF, DnaK, HchA, DnaJ, Spa, HslO, ClfB, SdrC, ScpA, SgtB, Atl MurA2	Michalik et al. (2017); Tartaglia et al. (2020); Li et al. (2022)

In addition to the characterization of pathogenic microorganisms, it may also be valuable to apply these techniques to the characterization of beneficial microorganisms involved in food processing. The fermentation process offers several benefits to food products, including enhanced flavor, improved taste, extended shelf life, and increased nutritional value. Fermentation is a biochemical process mediated by an array of enzymes, in which organic matter is transformed through microbial metabolism (Voidarou et al., 2021; Sun et al., 2022). The principal microorganisms involved in fermented foods are bacteria, molds, and yeasts. Within bacteria, *Bacillus*, *Bifidobacterium*, *Lactobacillus*, *Lactococcus*, *Leuconostoc*, *Pediococcus*, *Streptococcus*, and *Weissella* are the predominant genera of lactic acid bacteria (LAB) employed in the lactic acid fermentation of fermented milk products (Zapaśnik et al., 2022). Diverse biochemical reactions are initiated by multiple microorganisms during the fermentation, leading to the production of diverse metabolites with potential health benefits including vitamins, amino acids, enzymes, exopolysaccharides (EPS), short-chain fatty acids (SCFAs), organic acids, phenolic compounds, and bioactive peptides, among others. Many essential vitamins segregated serve as precursors of essential coenzymes and play a pivotal role in nucleic acid metabolism and post-synthesis protein modifications (Diez-Ozaeta and Astiazaran, 2022).

However, the process may also lead to the accumulation of undesirable and potentially toxic compounds. These include mycotoxins, bacterial toxins, biogenic amines, and cyanogenic glycosides (Sivamaruthi et al., 2019). Fermented dairy products and meat sausages are particularly susceptible to microbial contamination. Reports have identified bacterial toxins originating from *Bacillus cereus*, *E. coli*, and *Staphylococcus aureus* as significant contributors. These bacteria are associated with the production of emetic toxins, Shiga toxin (Stx) and Panton-Valentine leukocidin, respectively (Sivamaruthi et al., 2019). Biogenic amine-producing bacteria are typically part of the natural microbiota inhabiting animals and plants. These microorganisms include species from several well-characterized bacterial families, such as Enterobacteriaceae (e.g., *Escherichia* spp., *Klebsiella* spp., *Salmonella* spp.*, Shigella* spp.*, Proteus* spp.), Vibrionaceae, and *Pseudomonas*-like species (Abril et al., 2023). Those kinds of bacteria should be well characterized before being used in food products to avoid the development of nonspecific illnesses.

Among the most notable Gram-negative bacteria, we have focused on *Salmonella enterica*, *E. coli*, *Klebsiella pneumoniae*, *Shigella flexneri*, *Campylobacter jejuni, Pseudomonas aeruginosa*, and *Vibrio* spp. Regarding the most highlighted gram negative bacteria found in food products, *Salmonella* spp., *Escherichia* spp., *Klebsiella* spp., and *Shigella* spp. belong to the Enterobacteriaceae family.

*Salmonella* spp. is responsible for salmonellosis, the second most frequently reported foodborne gastrointestinal infection in humans, being also the four main causes of diarrheal diseases. Foods of animal origin, particularly pork, poultry, and poultry products such as eggs, are common sources of infection. S*almonella* genus is classified into two principal species: *S. enterica* and *Salmonella bongori*, and it is further characterized into more than 2,600 serotypes or serovars (Jajere, 2019). The typhoidal serotypes harbor specific virulence factors, such as the virulence CPS (Vi antigen) and the typhoid toxin, both linked to symptom development and immune evasion (Wang M. et al., 2020). *Salmonella* pathogenicity islands (SPIs) are distinct genomic regions within the bacterial chromosomes that encode a range of virulence factors (including those involved in adhesion, invasion, and toxin production), which are essential for infection and persistence. Many virulence markers contribute to pathogenesis, playing diverse roles in host interaction and disease progression. These include adhesion systems (adhesins, fimbriae, hemagglutinins, invasins, endotoxins, and exotoxins), capsule, flagella, plasmids, polysaccharides, and type III secretion systems (T3SS) encoded on SPIs (Andino and Hanning, 2015; Jajere, 2019; Pina et al., 2021; Morasi et al., 2022; Wójcicki et al., 2022; Chen K. et al., 2025) DNA microarrays have been developed in order to study *Salmonella* virulence. Huehn and Malorny (2009) designed a microarray incorporating 282 genetic loci from *Salmonella*. The array employed probes of 40–60 nucleotides to detect genes linked to serotype determination, virulence factors, prophage elements, fimbrial synthesis, and antimicrobial resistance. These microarrays have proven effective in elucidating the relationships and pathogenic potential of distinct *Salmonella* serotypes, as well as in revealing genetic variability among *Salmonella* Typhimurium strains (Hoorfar, 2011)Diverse authors investigated the proteome of different strains of *Salmonella* related to the virulence factors employing nLC-MS/MS technology. For instance, (Ferrer-Navarro et al., 2016), focused on the outer membrane proteins (OMPs), Saleh et al. (2019) studied the pathogenesis-related proteins of two *Salmonella* serovars (Typhimurium and Enteritidis) and Li Z. et al. (2019) compared the changes in the proteome of distinct host cells during infection with the serovar Typhimurium. Abril et al. (2025) performed an extensive proteomic analysis of various *Salmonella* serotypes from chicken meat. A variety of antimicrobial peptides (AMPs) were identified, including the Patatin-like phospholipase RssA, GDSL family lipases, TetR regulators, and penicillin-binding proteins. Host AMPs activate PbgA, which sustains the PhoPQ system, remodels the bacterial outer membrane, and enhances resistance to AMPs. Many virulence factors were identified, including, flagellar proteins (FliC, FlhE, FliT, FlhA, FliK), PI-2 (SSeC, SSeD, SSeE), toxins (VapC, HicA, HicB, HigA, HigB, ParD, YqcG, CcdB, ParD), and adhesins (Ag43, BigA, ShdA, FdeC, PilV, SfmA) among others. Moreover, Table 2 displays the recent studies for the virulence factors identified by MS of one of the most prevalent foodborne bacteria.

Moreover, *E. coli* is the most extensively studied bacteria within *Escherichia* genus. It is mainly found in undercooked meat, seafood, milk, vegetables, fruits, and water. It functions both as a gut commensal and as one of the most relevant human pathogens, capable of causing both intestinal and extraintestinal infections. The severity of the disease depends on the specific strain involved. In this way, several cases of gastroenteritis, bloody diarrhea, thrombotic thrombocytopenic purpura, and even neonatal meningitis have been reported. While most *E. coli* strains are harmless, some have evolved into pathogenic forms. These pathogenic strains are classified into two main categories based on their virulence factors: intestinal pathogenic *E. coli* (IPEC), and extraintestinal pathogenic *E. coli* (ExPEC). Foodborne illness outbreaks have been particularly linked to Shiga toxin (Stx) producing *E. coli* (STEC), which belongs to the IPEC category. Stx is the most potent biological cytotoxin encoded by a bacteriophage. ExPEC strains are defined by a diverse array of virulence factors such as toxins, capsules(adhesins, siderophores and invasins (Ramos et al., 2020; Sora et al., 2021; Singha et al., 2023).

Fagerquist et al. (2025) identified E8 and D colicins in 3 pathogenic STEC using MALDI-TOF-TOF technique. Through nLC-MS/MS, Wurpel et al. (2015) identified several distinct virulence-associated proteins including multiple adhesins (fimbriae), lipoproteins (ChuA, Lpp), and toxins (HlyA, CdtB), among others. The analysis of the extracellular vesicle proteome developed by Hong et al. (2019) employing nLC-TOF showed many proteins related with virulence, such as lipoproteins (Pal, RlpA), chaperonin GroEL, colicin, hemolysin HlyA, flagellar protein 3 FlgL, OMP OmpA, flagellin or Sat (Table 2). Comparing proteomics to DNA-based methods, a microarray capable of detecting 189 genes linked to virulence in *E. coli*, along with 30 genes conferring antimicrobial resistance, was developed (Hoorfar, 2011).

Furthermore*, Klebsiella* spp., is a widespread opportunistic bacterium responsible for a range of serious diseases in humans and animals, like bacteremia, bronchitis, meningitis, pneumonia, and urinary tract infections. Although it is more frequently linked to nosocomial infections, food (fish, fresh vegetables, raw food, juices, and milk) has also been identified as a potential route of transmission (Junaid et al., 2022; Alishvandi et al., 2025). Pathogenicity is largely driven by its ability to colonize host tissues, evade immune defenses, and cause cellular damage. These capabilities are primarily attributed to several key virulence factors (Alishvandi et al., 2025).

Huang et al. (2015) developed a MALDI-TOF MS based method to detect Hypervirulent *K. pneumoniae*. Magalhães et al. (2017) employing MALDI-TOF-TOF spectrometry compared the proteome profiles of multidrug-resistant (MDR) and susceptible clinical isolates identifying several virulence-proteins, including chaperones (DnaJ, GroEL), OmpA, CagA, SecA, MglB, TufA, TufB, RpoD, etc. Proteomic data of the research conducted by Illenseher et al. (2025) through nLC-MS/MS identified virulence factors such as OMPs (OmpA, OmpX), lipoproteins (Pal, SlyB), Wzx, fimbrial proteins (FimA_2, FimA_3), and chaperones (ClpB_2, DnaK_1, GroL_1 and HtpG). Fleitas et al. (2024) performed a label-free shotgun proteomic analysis to investigate the proteomic response of these bacteria to a synthetic antimicrobial peptide. Among the virulence-associated proteins identified using nLC-MS/MS, the PhoPQ (ArnA, ArnB, ArnC, ArnD, ArnT, LpxL, LpxO) and CpxRA system (Spy, CpxP, DsbA, DegP, PpiA) proteins were found, as well as proteins linked to catabolic processes (HutI, HutU, HutH, HutG, AstB, AstE, A6T8K5) (Table 2).

*Shigella* spp. comprises four species, with *S. flexneri* (serogroup B) and *S. sonnei* (serogroup D) being the most commonly detected. *Shigella* strains are highly effective at invading and replicating within human intestinal epithelial cells, leading to intense colon inflammation, also known as shigellosis or bacillary dysentery. These two serogroups occasionally produce Stx, which are AB5-type cytotoxin that inhibit protein synthesis, ultimately leading to cell death. The two variants, Stx1 and Stx2, are integrated into the bacterial chromosome (Sváb et al., 2017). Although the four species cause this infection, *S. flexneri* is the leading cause of endemic shigellosis. Foodborne outbreaks are commonly associated with fresh vegetables, unpasteurized milk, deli meat, ground meat, fish, and seafood, mainly due to insufficient thermal processing (Pakbin et al., 2021a; Zhu et al., 2021). The pathogenicity of this strain relies on the presence of a large virulent plasmid that encodes numerous components of T3SS. This includes the various proteins required to assemble the syringe-like Type III Secretion Apparatus (T3SA), along with transcriptional regulators, and their specific chaperones (Bajunaid et al., 2020; Zhu et al., 2021).

Various studies gather the protein profile of *S. flexneri*. For instance, Batalha et al. (2021) reported diverse virulence factors in *S. flexneri* strain 201 employing nLC-qTOF, including components of the S-adenosylmethionine cycle, exoenzyme AepA, lysozyme type C inhibitor YkfE and flavohemoprotein Hmp. Pettersen et al. (2021) investigated the proteomes of five *Shigella* strains by using label-free quantitative proteomics across nLC-MS/MS. Roughly 48 virulence-related proteins associated with adhesion (Iha), invasion (T3SS and T4SS), motility (PstB, PstC, PstS), and other functions were identified in each strain. Liu et al. (2017) identified the effector proteins secreted via the T3SS upon induction by chemical inducers throughout MALDI-TOF spectrometry, including IpaH1.4, IpaH_5, IpaH_7, IpaB, IpaC, OspD1, MxiC, and VirA, among others (Table 2).

*Campylobacter* spp. belongs to the family Campylobacteraceae, and it is among the four main global causes of gastroenteritis, with incidence rates rising steadily over the past decade. It is able to overcome the intestinal barrier achieving other tissues and organs, leading to other diseases like meningitis or endocarditis. Whiting this bacteria family, the great number of infections are attributed to *C. jejuni* and *Campylobacter coli* species (Costa and Iraola, 2019). They are commonly isolated from poultry and derived poultry products (Tegtmeyer et al., 2021). However, other additional sources include raw or undercooked meat, unwashed fruits and vegetables, raw milk, dairy products and contaminated water (Kreling et al., 2020). *Campylobacter* is recognized for its ability to form biofilms, which allows it to survive and adapt to environmental conditions (Laconi et al., 2023). The flagellum of these bacteria is crucial for pathogenesis, providing the necessary mobility and facilitating the penetration through the host intestinal mucin layer. They also have influence on chemotaxis, colony formation, virulence factors secretion, biofilm formation, and adhesion to the host cell which is accompanied by a strong pro-inflammatory immune response. Moreover, the cytolethal distending toxin (CDT), which induces DNA damage, has been identified in these bacteria (Kreling et al., 2020).

Focusing on *C. jejuni*, it is part of the normal intestinal microbiota in animals such as cattle, chickens, pigs, and sheep. Notably, the formation of biofilms plays a significant role in the environmental persistence and survival of this strain. Adhesion is a significant virulence mechanism that enables microbial colonization and promotes subsequent invasion (Kreling et al., 2020; Tikhomirova et al., 2024). Different researchers explored the protein profiles intimately related to the virulence factors of these bacteria through nLC-MS/MS technology (Tang et al., 2017; Taheri et al., 2019), iTRAQ labeling nLC-MS/MS (Sałamaszyńska-Guz et al., 2022), and MALDI-TOF MS (labeling nLC-MS/MS (Sałamaszyńska-Guz et al., 2022), and MALDI-TOF MS (Feucherolles et al., 2022).

*Pseudomonas* spp. belong to the family *Pseudomonadaceae*. It is commonly found in retail food products, including seafood and those derived from animals and plants. From the 313 species described, *P. aeruginosa* is the most clinically relevant (Bloomfield et al., 2024). Pathogenicity is associated with a wide array of virulence factors, including alginate, biofilm formation, Exotoxin A, flagellum, lipopolysaccharide (LPS), proteases, quorum sensing (QS), five secretion systems and type IV pili (T4P) (Rocha et al., 2019; Sauvage and Hardouin, 2020; Liao et al., 2022).

Several authors have contributed to proteomic study of this emphasizing the importance of virulence factors. Clamens et al. (2017) focused on amidase AmiE, which is involved in the regulation of *P. aeruginosa*, while Fléchard et al. (2018) investigated the alterations in the SigX protein, a factor involved in transcriptional regulation that affects membrane integrity and fluidity, both using nLC−MS/MS in proteomic analysis. Couto et al. (2015) analyzed the proteome of OMVs, and Ahmed et al. (2019) examined the effects of natural inhibitors on the repression of QS by nLC−MS/MS.

*Vibrio* spp. belong to the family *Vibrionaceae*. These genera are highly prevalent in aquatic environments and are associated with seawater fish, mollusks and crustaceans. Several species, such as *V. parahaemolyticus*, *V. vulnificus* and *V. cholerae* are well-known enteric pathogens in human (Mougin et al., 2020). *V. parahaemolyticus* is the major seafood-borne pathogen responsible for gastroenteritis, septicemia, and severe wound infections. Its pathogenicity is attributed to a range of virulence-associated factors, including hemolysins, secretion systems (T3SS, T6SS), adhesion molecules, iron acquisition systems, surface polysaccharides, proteases, and OMPs such as LptD (Li L. et al., 2019). *V. vulnificus* infection typically occurs through the consumption of contaminated seafood, particularly raw oysters. Additionally, it can infect certain fish species, leading to diseases characterized by hemorrhagic septicemia and death. It is considered one of the most life-threatening due to its high mortality rate. Its pathogenicity is due mainly to the production of several exotoxins, including the multifunctional autoprocessing repeats-in-toxin (MARTX), phospholipase A2, the metalloprotease elastolytic, and the pore-forming toxin VvhA. Biofilm-associated factors also play a significant role in the virulence. The components involved in the colonization and persistence of this bacteria are flagellins, CPS, exopolysaccharides (rbd, brp, and EPS-III locus), and calcium-binding protein (CabA). Another major virulence factor HlyU, a key transcriptional regulator (Choi and Choi, 2022). Regarding *Vibrio cholerae*, it is responsible for widespread outbreaks and global epidemics of cholera. The cholera toxin (CT) is the major virulence factor and it is secreted by the type II secretion system (Telesmanich et al., 2024).

Zoued et al. (2021) performed a proteomic analysis of rabbits infected with wild-type *V. cholerae* using MS3 spectrometry of labeled peptides with TMT reagent. They identified a set of virulence components, including OMPs (OmpA, OmpU, OmpV, OmpT, OmpW), flagelar proteins (FlgE, Flgk, FlgL), chaperones (GroEL, DnaK), toxin co-regulated pilus (TcpB, TcpC, TcpD, TcpF, TcpP, TcpQ), cholera toxin subunit A (CtxA), accessory colonization factor (AcfA) and the outer membrane heme receptor HutA among others (Table 2). Yang et al. (2015) conducted a detailed proteomic analysis of *V. parahaemolyticus* labeled proteins using iTRAQ labeling, exposed to various culture conditions and separated and identified with nLC- TOF MS. A significant number of proteins were identified associated with the outer membrane (OmpA, OmpK, OmpU, TolC), iron-regulation (FeoB, Fe-S protein NrfC), transport (HutA, CorA, ExbB, ScrB), adhesins (MshA, Tfp), T2SS, T3SS (YscC, YscL), T5SS (ClpV1, VC_A0110 family), chaperone (DnaK), hemolysins (GN VP0730, VPA0257, VP2536), and regulatory proteins (GntR, Hfq, LuxR, rsmA, VpsT) (Table 2). Heinz et al. (2022) analyzed the stressosome of *V. vulnificus*, a stress-sensing protein complex assessed by nLC-Ms/MS. They identified numerous proteins linked to pathogenicity like RtxA adhesin, flagellin FliC, various flagellar components (*flgA*, *flgB*, *flgE*, *flgF*, *flgL*, *flgK*, *flgJ*), Rtx, and several chaperones (DnaK, DnaJ, ClpB, ProQ). Other researchers conducted a comparative proteomic analysis of OMP changes in *V. vulnificus* after stimulation with sewage. They reported a variety of virulence proteins such as EF-Tu, MotB, HupA, OmpN, OmpU, and Trx through MALDI-TOF technique (Zhang et al., 2017) (Table 2).

Among the most notable Gram-positive bacteria, we have focused on *L. monocytogenes*, *B. cereus, Bacillus licheniformis*, and *S. aureus*.

The *Listeria* genus belongs to the family *Listeriaceae*. *Listeria* species are capable of colonizing a diverse array of environments and they are frequently isolated from various food products, such as milk and dairy products, RTE food, poultry, fungi, fruits and raw meat. *Listeria* comprises 26 species, however, *L. monocytogenes* and *Listeria ivannovi* are considered pathogenic (Rossi et al., 2022). The pathogenicity of *L. monocytogenes* is attributed to several virulence factors, which are regulated by the transcriptional factor PrfA. These factors include the pore-forming toxin Listeriolysin O, Internalins InlA and InlB, which are critical for bacteria penetration into host cells, Phospholipases PlcA and PlcB, ActA that enables the intracellular motility, metalloprotease (Mpl), and UhpT that promotes proliferation. *Listeria* pathogenicity island (LIPI) 1 plays a critical role in the infection process and survival of the strain (Erol and Taşçi, 2021; Zhang et al., 2024).

Luciani et al. (2025) identified various non-cytoplasmic proteins associated with virulence during the exponential growth phase under different conditions using nLC-MS/MS. Several of these proteins were linked to flagellar assembly (FlgK, FlgE, FlgL, MotB, Lmo0696, Lmo0706), adherence (Lmo072, OpuCA, pbpA, ClpP), cell Wall (LPxTG, Lmo0129). Abril et al. (2021) identified bacterial peptides corresponding to specific virulence factors through shotgun proteomics (using nLC-MS/MS) in 9 strains of *Listeria*. Over 395 virulence factors were reported. They included transcriptional regulators (MarR, TetR), toxins (HiA, LGX, RelE, ParE, PemK, MazF), colonization (EssA, EssB, VirB4, Mga, ESAT-6, GtcA, P60, M60, ComGA, ClpX, ClpP, ClpY, InlA, InlB, InlC, InlJ). Stincone et al. (2020) conducted a comparative analysis of peripheral cell component (PCC) proteins of *L. monocytogenes* exposed to a sub-lethal concentration of nisin. Key virulence factors identified by nLC-MS/MS included Listeriolysin O, *Listeria* adhesion protein (LAP), actin assembly-inducing protein (ACTA) and internalin B (InlB). Additional virulence proteins were related to biofilm formation (FlaA, FlgE, FruA, PdhB, Lmo1371), transport (Lmo2196, Lmo0135) and folding (PrsA) (Table 2).

The genus *Staphylococcus*, which belongs to the family *Staphylococcaceae*, includes over 50 species that are commonly found as colonizers of the skin and mucous membranes in a wide range of animal species. Among them, *S. aureus* stands out as a major pathogen, responsible for numerous infections in both humans and animals, and is recognized as a leading cause of foodborne illnesses worldwide (Fetsch and Johler, 2018). It has been detected in milk, dairy products, poultry, meat products, and fishery products. Furthermore, it is a primary contributor to mastitis in cattle, goats, and sheep, resulting in reduced milk production and significant economic losses. The remarkable stability, along with its resistance to key proteolytic enzymes such as pepsin and trypsin, ensures that the toxins retain their emetic activity within the gastrointestinal tract (Pal et al., 2021). The primary virulence factors include enzymes (coagulase, DNase, hyaluronidase, lipase, staphylokinase), structural components (capsules, collagen, fibrinogen, elastin binding proteins) and exotoxins (Abril et al., 2020). The emergence and persistence of multidrug-resistant *S*. *aureus* (especially Methicillin-resistant *S. aureus*) in hospitals represents a major public health challenge, requiring coordinated infection control measures, surveillance, and responsible antibiotic use. Momoh-Zekeri et al., have developed a microarray kit that encompasses 334 specific sequences, including markers for *S. aureus* species, genes linked to antimicrobial resistance, loci encoding staphylococcal enterotoxins (SEs) and enterotoxin-like proteins, elements of the accessory gene regulator system, as well as markers associated with capsule formation and biofilm development, alongside a broad range of additional genetic targets (Momoh-Zekeri et al., 2021).

Regarding proteomics. several studies of *S.aureus* have been performed using nLC-MS/MS analysis The investigation of Li et al. (2022) on the use of an antibacterial species in sauced beef revealed a variety of virulence proteins such as adhesins (ClfA, ClfB, SraP), regulators (CodY, SarR, Fur, Rot), or lipoproteins (Lpl9, AdcA). Tartaglia et al. (2020) reported the protein content of extracellular vesicles from *S. aureus*. Over 92 virulence proteins were identified, including those related to biofilm formation (Nuc), adhesion (EbpS, Eno, Map_2), evasion (PsmB1, PsmB2, Sbi, HlyII, LukF, LukG, LukH), regulation (AgrD), toxins (PsmA1-PsmA4, Hld, HlyII), and lipoproteins (MetQ, PhnD, PstS, OppA, OpuCC, PrsA, Lpl14, YidC, BdbD). Michalik et al. (2017) performed a DIA-MS analysis to investigate strain adaptation in epithelial cells. They identified a large number of pathogen proteins, including chaperones (DnaK, HchA, DnaJ) and siderophores (SbnA, SbnC, SbnE, SbnF), as well as others involved in various functions such as regulation (SigB, SpoVG, CcpA, CtsR, FmtA, AgrA, Rot), folding (PrsA, clpB), adhesion (Spa, HslO, ClfB, SdrC), and capsule (ScpA, SgtB, Atl MurA2) (Table 2).

Species of the genus *Bacillus,* belonging to the *Bacillaceae* family, are spore- and biofilm-forming bacteria commonly found in nature. They are frequently isolated from both fermented and unfermented food and feed products. Several strains have been evaluated for their probiotic potential and have been patented for use in health supplements. These strains have demonstrated capabilities in pathogen exclusion, antioxidant activity, immunomodulation, and food fermentation. Despite these benefits, *Bacillus* species are opportunistic pathogens that can cause serious local or systemic infections, such as endophthalmitis and septicemia, when they gain access to mammalian tissues, specially *B. cereus*, for producing the emetic toxin Cereulide (Hu et al., 2021). Gao et al. (2021) reported that the vegetative cells *B cereus* are capable of producing both enterotoxins and membrane-damaging toxins identified using nLC-MS/MS TOF spectrometer. Upon ingestion, the toxins secreted can cause either diarrheal or emetic syndromes *B. licheniformis* has been identified as a major microbial contaminant in milk powder production facilities. Numerous isolates have been reported to contribute to dairy spoilage due to their ability to secrete enzymes such as β-galactosidases, lipases and other proteases analysed by nLC-MS/MS of labeled peptides with TMT reagent (Wang N. et al. (2020).

The characterization of the pathogenic mechanisms of foodborne bacteria through shotgun proteomics and related techniques, conducted prior to the commercialization of food products, could play a pivotal role in preventing the emergence of foodborne diseases associated with the food industry. Furthermore, the application of these techniques to food samples or clinical isolates could enable the rapid identification of the most effective chemotherapeutic agents and provide insights into the potential progression of the disease, based on comparative analyses with previously characterized strains exhibiting similar pathogenic profiles or virulence factors.

## 4 Shotgun proteomics for foodborne pathogenic bacteria identification biomarkers

Shotgun proteomics has enabled to characterize numerous foodborne pathogens. Among these detected proteins as described above, species- or strain-specific peptides can be found, allowing us to identify them in complex food simples. The concept of proteotyping—the identification and typing of pathogens based on proteomic fingerprints—has greatly benefited from advances in MS/MS (Karlsson et al., 2017; Karlsson et al., 2020). These techniques allow for species- and even strain-level resolution, which is critical in outbreak tracking and source attribution.

The selection of putative peptide biomarkers is carried out through a large computer-assisted proteomic comparison with known protein databases; the specificity of the peptides identified; by LC-ESI-MS/MS, in only one of the species analyzed are subsequently verified by sequence homology using the BLASTp algorithm. Peptides that matched with a unique species in the GenBank database (or/and other databases) are proposed as species-specific tryptic peptide biomarkers.

The application of high-throughput LC-ESI-MS/MS in shotgun proteomics has facilitated the identification of species-unique peptide biomarkers for pathogens such as *S*. *aureus*, *S*. *enterica, L*. *monocytogenes*, *Enterococcus faecium*, *Streptococcus* spp. from dairy products (Abril et al., 2020), and various *Pseudomonas* spp. isolated from food matrices like meat, dairy, and seafood (Carrera et al., 2017; Abril et al., 2024a; Abril et al., 2025). For instance, Carrera et al. (2017) demonstrated that species-specific peptide signatures could reliably differentiate strains of *S. aureus*, correlating their proteomic profiles with functional networks involving virulence and host-pathogen interaction pathways. In the context of *Salmonell*a, specific taxon and serovar-level peptide markers have been elucidated and validated (Chen S. H. et al., 2019; Chen et al., 2021; Abril et al., 2025), facilitating precise identification that surpasses conventional culture-based methods.

In dairy products, shotgun proteomics has successfully been applied to detect and differentiate *Enterococcus* species, revealing virulence-associated proteins and bacteriocin biosynthesis pathways, which are crucial for understanding both their probiotic potential and pathogenic risks (Quintela-Baluja et al., 2022; Abril et al., 2022).

The most studied applications include seafood and dairy food matrices. Seafood represents a high-risk food matrix due to its susceptibility to contamination by biogenic-amine-producing and spoilage bacteria. Recent studies have leveraged shotgun proteomics to perform quantitative and comparative proteomic analyses of such microbiota, uncovering virulence determinants, amine biosynthetic enzymes, and proteins associated with resistance to environmental stresses in biogenic amine bacteria from seafood (Abril et al., 2023; Abril et al., 2024b). These efforts have identified peptide biomarkers that are not only diagnostic but also suggest targets for food preservation strategies.

Emerging methodologies now integrate shotgun with targeted proteomics for increased sensitivity and specificity. For example, tandem mass tag (TMT)-based approaches have uncovered regulators of biofilm formation in *B. licheniformis*, a spoilage organism of food concern (Wang N. et al., 2020), while selected reaction monitoring (SRM) and label-free quantitation have been employed for precise identification of pathogens in juices and leafy greens (Chen C. T. et al., 2019; Chen M. et al., 2025). Additionally, machine learning and *in silico* peptide mass library tools have expanded the potential for rapid and automated detection (Lasch et al., 2020; Mappa et al., 2023), enhancing reproducibility and throughput in food safety surveillance.

**FIGURE 2:**
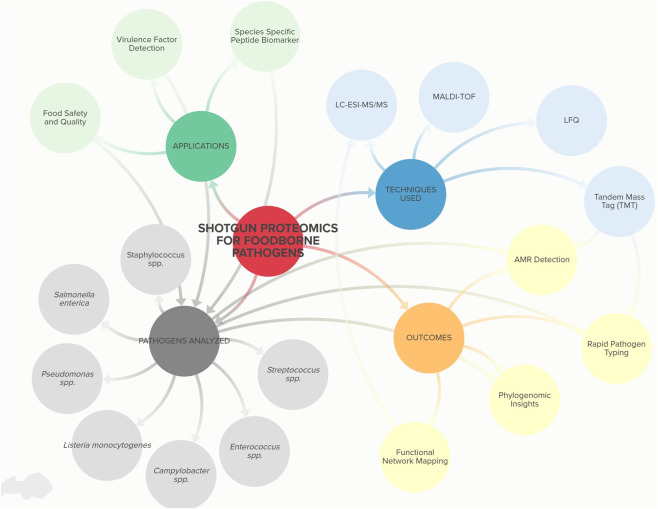
Bibliometric network illustrates the main applications and methodologies of shotgun proteomics in foodborne pathogens research. The central node represents the main theme: *Shotgun proteomics for foodborne pathogens*. Surrounding branches illustrate key components: Pathogens analyzed (green), including *Pseudomonas* spp., *Streptococcus* spp., *Salmonella enterica*, *Enterococcus* spp., *Listeria monocytogenes*, *Staphylococcus* spp., and *Campylobacter* spp.; Techniques used (purple), such as MALDI-TOF, label-free quantification (LFQ), LC-ESI-MS/MS, and tandem mass tag (TMT); Applications (yellow), including food safety and quality assessment, virulence factor detection, and species-specific peptide biomarkers; and Outcomes (pink), encompassing functional network mapping, antimicrobial resistance (AMR) detection, rapid pathogen typing, and phylogenomic insights.

Table 3 displays an overview of putative biomarker determination by shotgun proteomics applications in foodborne pathogen. The elucidation and evaluation of these peptide biomarkers, and they use in targeted proteomics provide a fast and accurate identification of pathogen strains in complex foodstuffs in less than 2 h. Figure 2 illustrates the main applications and methodologies of shotgun proteomics in foodborne pathogens research including characterization, identification and detection.

**TABLE 3:** Overview of putative biomarker determination by shotgun proteomics applications in foodborne pathogen.

Pathogen	Matrix food sources	Key findings protein biomarkers identified	Applications in detection	Studies (APA abbreviated)
*Staphylococcus aureus*	Foodborne strains; dairy products	644 non-redundant proteins identified; species-specific peptide biomarkers; virulence factors including enterotoxins; bacteriophage peptides	Strain-level differentiation; identification of virulence profiles; phage-host interaction analysis	Carrera et al. (2017); Abril et al. (2021)
*Streptococcus* spp.	Milk from mastitis cases	1,890 proteins identified; 65 phage-origin peptides specific to *Streptococcus* spp.	Phage characterization; pathogen typing	Abril et al. (2020)
*Enterococcus faecium*	Dairy and fermented food products	1,327 proteins identified; bacteriocin-associated peptides; virulence factors including adhesins and antibiotic resistance proteins	Rapid strain-level identification; food safety assessment	Quintela-Baluja et al. (2022); Abril et al. (2022)
Biogenic-amine-producing bacteria (e.g., *Morganella*, *Photobacterium*)	Seafood	1,811 proteins identified; enzymes involved in histamine, cadaverine, tyramine, and putrescine synthesis	Spoilage and safety risk assessment in seafood	Abril et al. (2023); Abril et al. (2024a)
*Pseudomonas* spp.	Fish products	Spoilage-associated enzymes (e.g., proteases); biofilm-related proteins	Quality assessment and control in fish industry	Abril et al. (2024a)
*Salmonella enterica*	Chicken meat	Virulence-related proteins; peptide markers for serotyping	Serovar differentiation; targeted MS detection	Abril et al. (2025); Chen et al. (2025a), Wang et al. (2022b); Chen et al. (2021); Chen et al. (2019b)
*Cronobacter turicensis*	Powdered infant formula	OMPs; iron uptake proteins	Strain-level proteomic profiling	Carranza et al. (2009)
*Listeria monocytogenes*	Biofilm cultures at various temperatures	Temperature-dependent stress proteins; biofilm adaptation markers	Adaptation and virulence assessment	Santos et al. (2017); Santos et al. (2019)
Various foodborne pathogens (e.g., *E. coli*, *Salmonella*, *Listeria*)	Meat, juice, lettuce	Species-specific tryptic peptides	Rapid identification using selected reaction monitoring (SRM) or magnetic nanoparticles	Chen et al. (2019a), Gekenidis et al. (2014)
*Bacillus licheniformis*	Dairy/environmental biofilms	Biofilm formation regulators (e.g., Spo0A, ComX)	Biofilm control strategies in food plants	Wang et al. (2020b)

## 5 Shotgun proteomics for allergen detection in food

Food allergy is caused by an immune reaction to protein antigens in food, known as “allergens” (Dramburg et al., 2023; Sicherer and Sampson, 2018). Exposure to the allergen, even in trace amounts, can trigger a wide range of symptoms (e.g., gastrointestinal disorders, airway inflammation, anaphylaxis), whereas non-allergic individuals experience no adverse effects. Despite the lack of precise epidemiological data, food allergies are estimated to affect 6%–8% of young children and 3%–4% of adults, but their prevalence appears to be increasing (Dramburg et al., 2023; Sicherer and Sampson, 2018). Plus, there is no treatment to food allergy but the strict avoidance of allergenic proteins. For these reasons, food allergy is becoming a serious issue worldwide, highlighting the pressing need for improved strategies in prevention and management.

In this context, MS-based proteomics has emerged as a key approach for the detection and detailed analysis of allergenic proteins and the design of effective control measures. Its enhanced sensitivity and specificity offers the potential for more accurate, reliable, and scalable methods in both research and routine testing (Marzano et al., 2020; Carrera et al., 2024; Koeberl et al., 2014).

### 5.1 Precise identification and quantification of allergens

Although regulations have been established for the intentional addition of allergenic ingredients (Chang et al., 2023), they do not account for potential cross-contamination during processing and they do not define threshold doses that trigger allergic reactions. For this reason, Precautionary Allergen Labelling (PAL) has been implemented to warn consumers about the possible presence of undeclared allergens in food products (DunnGalvin et al., 2015). However, PAL is rarely based on risk assessments and can confuse allergic consumers, leading them to avoid products that may not actually contain the indicated allergen (Bugyi et al., 2023). To address this issue, efforts have been made to establish reference doses (Westerhout et al., 2019; Remington et al., 2020). To date, the most recognized framework is VITAL (Voluntary Incidental Trace Allergen Labelling) project by Allergen Bureau of Australia and New Zealand (Taylor et al., 2018). VITAL panel sets allergen concentrations below which PAL is unnecessary, based on updated clinical data. Therefore, methodologies for allergen detection and quantification must take these levels into account.

So far, ELISA has been the most widely used method for allergen detection. However, its limitations have been repeatedly highlighted, including false positives, reduced accuracy in complex food matrices, challenges in multiplex testing, reliance on specific antibodies and cross-reactivity, among others (Anđelković et al., 2015; Ruethers et al., 2020; Stelk et al., 2013). Currently, LC-MS technologies are emerging as a promising tool for the identification and quantification of food allergens, due to their high sensitivity, robustness, and capability for high-throughput analysis on a large-scale (Marzano et al., 2020; Carrera et al., 2024; Koeberl et al., 2014). Moreover, their suitability for detecting allergens at VITAL threshold levels has been demonstrated (Holzhauser et al., 2020).

**FIGURE 3:**
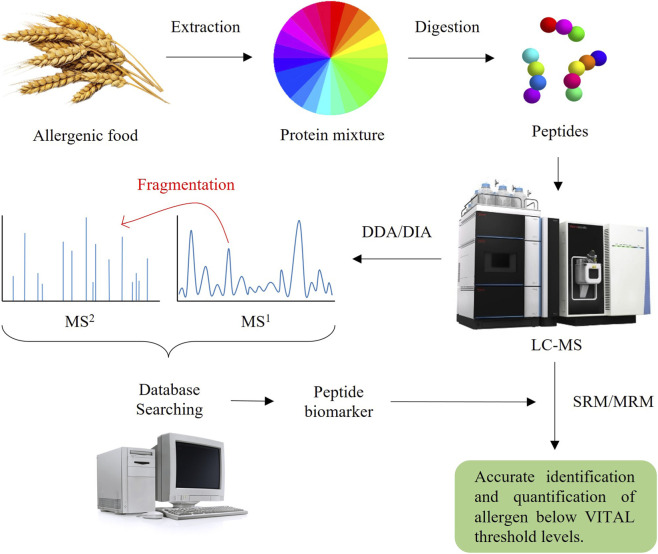
General workflow for allergen identification and quantification. The protein mixture obtained from the sample is digested, and the resulting peptides are subjected to MS analysis. Discovery approaches (DDA/DIA) are used to identify candidate peptide biomarkers, which are then employed in Targeted methods (SRM/MRM) to detect and quantify the allergen at VITAL threshold levels.

As previously discussed, shotgun proteomics is the most commonly used approach for biomarker identification and quantification. The general workflow is illustrated in Figure 3, serving as a basis from which numerous analytical methods have been developed to enable the reliable detection of major food allergens. A label-free quantification approach was used to select target peptides from six different commercial soy ingredients, enabling their unambiguous identification and consistent detection using a Parallel Reaction Monitoring (PRM)-based approach (Chen S. et al., 2019). Another study developed and validated a Multiple Reaction Monitoring (MRM) method on a triple quadrupole platform for sensitive detection of milk and egg proteins in processed foods, successfully identifying trace amounts below 0.2 mg (Monaci et al., 2020). By using Stable 1sotope-Labeled (SIL) peptides as internal standard, quantification of seven sesame allergen proteins with high specificity and reproducibility was achieved (Ma et al., 2020). A rapid method for detecting fish β-parvalbumins in food products was developed by our group, combining heat-based protein purification, ultrasonic-assisted trypsin digestion and targeted monitoring of 19 peptide biomarkers using selected MS/MS ion monitoring (SMIM) on a linear ion trap mass spectrometer. The results could be achieved in under 2 hours, highlighting the method’s high potential for rapid allergen screening in food safety applications (Carrera et al., 2012).

High-resolution (HR) mass spectrometers are increasingly being used for allergen detection, since they provide enhanced selectivity due to mass accuracy. This study compared different acquisition modes for the identification of five allergenic ingredients using a HR hybrid quadrupole-Orbitrap platform. Targeted selected ion monitoring with data-dependent fragmentation (t-SIM/dd2) proved to be the most effective approach, enabling the simultaneous detection of 17 peptides in a single run (Pilolli et al., 2018). Another method using a HR-Orbitrap mass spectrometer was developed to detect peanut contamination in complex nut mixtures, achieving detection limits as low as 4 µg of peanuts proteins (Monaci et al., 2015). A strategy using QTOF-based LC-MS for the simultaneous detection and quantification of almond, cashew, peanut, and walnut allergens in bakery products, achieved high sensitivity, specificity, and validation against VITAL reference doses (Xu et al., 2024).

Moreover, MS technologies have the potential to enable the simultaneous detection of multiple allergens from diverse sources. Efforts are being made on this approach, since it is particularly suitable for routine laboratories (where using a specific method for each sample is often impractical). In this study, a UHPLC–MS/MS-based method was developed to detect ten different allergens in complex and processed food matrices, achieving accurate quantification at low concentration levels (Planque et al., 2017). Another LC–MS/MS method for the simultaneous detection of 13 allergenic food ingredients demonstrated its effectiveness in both raw and thermally processed foods (Ogura et al., 2019). To facilitate multiplexing, concatemerized SIL peptides are currently under development. A study demonstrated that a^15^N-labeled concatemer containing multiple signature peptides can serve as an effective internal standard for allergen quantification in processed food matrices with egg, milk, peanut, and hazelnut (Gavage et al., 2020). Nonetheless, the wide diversity of allergens, food matrices, and processing conditions can restrict simultaneous methods to analyzing food products with similar physicochemical properties (Pedreschi et al., 2012).

Recent advances in LC-MS technology focus on enhancing analytical performance. Emerging research is exploring MS^3^ as a strategy to further improve sensitivity through an additional stage of fragmentation (Brockmeyer, 2018). This study employed MRM cubed (MRM^3^) technology to achieve highly sensitive and specific detection of six tree nut allergens across various food matrices, demonstrating up to 30-fold increased sensitivity over conventional MRM approaches (Korte and Brockmeyer, 2016). Another approach using MRM^3^ allow the detection of crustacean allergens down to concentrations of 25 μg_allergen_/g_food_ (Korte et al., 2016). However, this approach lengthens cycle time, limiting the use of multianalyte methods with many peptide markers.

Despite its advantages, one of the main limitations of MS technology lies in the complexity of food matrices, which can interfere with subsequent analyses (Hu et al., 2024). Consequently, various methods focus on improving sample preparation. A novel approach employing immunomagnetic nanoparticles (IMNPs) to extract mango allergens in processed foods was useful to enhance MS analysis (Tsai et al., 2024). An innovative strategy combined ELISA-based peptide enrichment prior to MS analysis, enhancing the sensitivity for detecting the egg allergen Gal d 2 (Röder et al., 2021). Another approach combines a food extraction solution (ExSta™) and a pre-treatment kit (EasyPep™) to improve allergen extraction (Oyama et al., 2024). It is also interesting to optimize sample preparation workflows for specific complex matrices, such as chocolate (Henrottin et al., 2023).

Overall, these precise allergen detection techniques can serve as effective tools for detecting allergens below VITAL threshold doses, thereby avoiding unnecessary labeling. However, their routine adoption in testing laboratories remains limited, highlighting the need for further development to enable high-throughput applications.

### 5.2 Allergen characterization

In addition to allergen detection, shotgun proteomics can be applied to gain deeper insights into their sequence, structure and properties. In our lab, full *de novo* sequencing of 25 new β-parvalbumin isoforms from *Merluciidae* family was carried out with a novel strategy combining two dimensional gel electrophoresis (2-DE) bottom-up proteomics with Fourier-transform ion cyclotron resonance MS (FTICR-MS) and SMIM (Carrera et al., 2010). The complete sequences were deposited in UniProtKB and Allergome databases (accession numbers: P86739–P86775). As an extension of their sequencing power, MS-based technologies can provide information about isoforms and PTMs. Since these features have been shown to modulate protein allergenicity, their evaluation is crucial. Using a combination of bottom-up, middle-down, and top-down proteomics, this study revealed extensive sequence heterogeneity in the mustard allergen Sin a 1, identifying 24 polymorphisms and eight isoforms (Hummel et al., 2015). By analyzing salmon parvalbumins with MALDI-TOF and ELISA, it was demonstrated that the two isoforms (Sal s 1 beta 1 and Sal s 1 beta 2) exhibit different allergenic properties (Perez-Gordo et al., 2012).

These advanced proteomic approaches enable the identification of structural features relevant to immunogenicity and, consequently, support the design of hypoallergenic variants suitable for use in immunotherapy (Cook and Burks, 2018). As an example, B-cell epitopes of β-parvalbumins from various fish species were characterized in our group, identifying potential peptide vaccine candidates for fish allergy (Carrera et al., 2019). To develop such therapeutics, methods for recombinant allergen production in bacteria and yeast have been proposed (Lin and Alcocer, 2017).

### 5.3 Food processing assessment

Various food processing techniques are widely applied to improve the safety, shelf life and sensory qualities of food products. However, these processes can also significantly alter the structure and biochemical properties of food proteins, hence affecting its allergenicity (Verhoeckx et al., 2015). Thermal processing has been shown to decrease protein allergenicity through degradation or denaturation, or even increase it through the formation or exposure of neoepitopes (Pi et al., 2024).

MS techniques have been widely applied to gain insights into this issue due to their ability to detect and quantify allergens, as well as to identify PTMs. Effects of high-pressure treatment on the muscle proteome of hake was evaluated by 2-DE and LC-MS/MS, showing that the abundance of β-parvalbumin increased after treatment (Carrera et al., 2018). More than 40 PTM modifications were identified by LC-Orbitrap when studying the effect of roasting on peanuts (Đukić et al., 2022). Moreover, food processing may reduce allergen detectability, as suggested by this study, which reported a 20%–83% signal loss after baking bread and cookies (Korte et al., 2019).

## 6 Future applications of shotgun proteomics

The future of shotgun proteomics in food safety lies both in the development of new research applications for identifying biomarkers to detect emerging microbial pathogens and allergens in foods, and in its integration into routine monitoring and real-time control systems used by the food industry and regulatory agencies. As MS technologies continue to advance, improvements in sensitivity, speed, and cost-effectiveness are expected to make high-throughput proteomic analyses more widely accessible.

Several expected future applications of shotgun proteomics in this field include:

- High-throughput Detection Systems: Shotgun proteomics may support the development of multiplex detection systems for simultaneously identifying a broad range of pathogens and allergens in a single analyze. This capability is particularly important for complex food matrices where multiple contaminants may be present. These platforms can be designed to detect unique peptide biomarkers from various species or allergenic sources.
- Real-Time Pathogen Monitoring: Progress in portable MS and on-site sample processing technologies may enable the use of shotgun proteomics on site. Future devices—such as benchtop instruments—could carry out real-time proteomic analyses to rapidly identify contaminants during the farm-to-fork chain.
- Identification of Novel Food Pathogens and Allergens: Shotgun proteomics should play a critical role in advancing allergen risk assessment due to the differentiation of allergenic and non-allergenic variants of similar proteins and improve labeling accuracy and the identification of novel pathogens and allergens in emerging food sources such as plant-based proteins, insects, and alternative foods and those driven by climate change.
- Data-Driven Predictive Modelling: Integrating shotgun proteomics with ML and AI should support the development of predictive models for contamination risks. By correlating proteomic data with environmental conditions, these models can forecast the likelihood of pathogen outbreaks or allergen presence, supporting targeted sampling and proactive interventions.
- Integration with Other Omics Technologies: The future should improve the integration of shotgun proteomics with other omics approaches, such as genomics, transcriptomics, and metabolomics, achieving a systems biology perspective and a better comprehensive understanding of microbial activity and allergenic potential in food environments.
- Customizable Proteomic Kits: Commercialization of ready to use proteomic kits for specific pathogens, or allergens represents another promising application.
- Regulatory and Industry Implementation: The industry and regulatory institutions may begin incorporating these methods into official food safety protocols.

Therefore, shotgun proteomics is expected to evolve from a primarily research-focused tool into a methodology relevant for the control of food safety during the whole food chain. The development of faster, more accurate, and comprehensive shotgun proteomics-based approaches for the detection of microbial pathogens and allergens is expected to be relevant in the future. To achieve this relevant situation, improvements in sample preparation, instrumentation, data analysis, and integrative omics will be crucial. Moreover, shotgun proteomics should have a relevant role anticipating and adapting to emerging food safety threats, such as those intensified by climate change, and maintaining a resilient and secure global food supply.

## 7 Concluding remarks

MS plays a principal role in shotgun proteomics providing high sensitivity, accuracy, and resolution for peptide detection and sequencing (Shuken, 2023). Various fragmentation techniques have emerged improving sequence coverage and enhancing the identification of PTMs (Brodbelt, 2014). Data acquisition strategies in shotgun proteomics have also evolved significantly. Quantification in shotgun proteomics can be achieved through label-free methods or labeling strategies. In food science, these techniques and omics data integration help to characterize microbial contamination, allergen presence, and food matrix changes more thoroughly.

Shotgun proteomics has emerged as a powerful tool for identifying species-specific biomarkers linked to foodborne pathogenic bacteria, including those involved in virulence, antibiotic resistance (Abril et al., 2024a; Abril et al., 2025). These molecular markers enhance the ability to detect pathogens and provide deeper insights into their biological behavior and mechanisms of pathogenicity. Among the most notable bacteria found in food products such as *L. monocytogenes, B. cereus, B. licheniformis, S. aureus, S. enterica, E. coli, K. pneumoniae, S. flexneri, C. jejuni, P. aeruginosa*, and *Vibrio* spp., several studies investigated the proteome of different strains including the presence and characterization of virulence factors and antimicrobial resistances. This characterization, along with the identification of specific biomarkers of pathogens isolated from food that have caused infections in individuals, will allow the rapid identification of the most effective antimicrobial for each pathogen and prevent the emergence of foodborne diseases associated with the food industry. The elucidation and evaluation of these peptide biomarkers, and their use in targeted proteomics provide a fast and accurate identification of pathogen strains in complex foodstuffs in less than 2 h.

Food allergies are an increasingly significant global concern, underscoring the urgent demand for better preventive and management strategies. To date, ELISA has been the most commonly employed technique for allergen detection; however, it is associated with several significant limitations (Anđelković et al., 2015; Ruethers et al., 2020; Stelk et al., 2013). In this regard, shotgun proteomics has significantly improved the detection of food allergens by enabling the direct identification of allergenic proteins within complex food samples, addressing several limitations inherent to traditional immunoassay-based methods (Carrera et al., 2024). Studies have focused on allergens from a variety of sources, including peanut, fish, milk, eggs, sesame, and hazelnut, among others, as well as crustacea, due to their prevalence in allergic reactions.

Moreover, MS technologies offer the potential for the simultaneous detection of multiple allergens originating from diverse sources, both in raw and thermally processed food products (Ogura et al., 2019). These advanced techniques not only allow for the precise identification and quantification of allergens but also provide deeper insights into their amino acid sequences, structural conformations, and biochemical properties, including the detection of isoforms with varying degrees of allergenicity. Importantly, MS approaches facilitate the assessment of food processing effects, as thermal treatments and other processing methods can significantly alter the structure and biochemical characteristics of proteins, potentially modifying their allergenic potential.

MS technologies continue to advance, improvements in sensitivity, speed, and cost-effectiveness are expected to make high-throughput proteomic analyses more widely accessible. In this sense, several anticipated future applications of shotgun proteomics in this field include: (i) High-throughput detection platforms capable of simultaneously identifying a broad range of pathogens and allergens in a single assay; (ii) Real-Time Pathogen Monitoring as portable MS and on-site sample processing technologies; (iii) identification of novel pathogens and allergens in emerging food sources; (iv) Data-Driven Predictive Modeling: Integrating shotgun proteomics with ML and AI; (v) Integration with Other Omics Technologies; (vi) Customizable Proteomic Kits; and (vii) Regulatory and Industry Implementation.
